# How Do Children Restrict Their Linguistic Generalizations? An (Un-)Grammaticality Judgment Study

**DOI:** 10.1111/cogs.12018

**Published:** 2012-12-18

**Authors:** Ben Ambridge

**Affiliations:** Institute of Psychology, Health & Society, University of Liverpool

**Keywords:** Child language acquisition, *Un*- prefixation, Derivational morphology, Retreat from overgeneralization, Pre-emption, Entrenchment, Verb semantics

## Abstract

A paradox at the heart of language acquisition research is that, to achieve adult-like competence, children must acquire the ability to generalize verbs into non-attested structures, while avoiding utterances that are deemed ungrammatical by native speakers. For example, children must learn that, to denote the reversal of an action, *un*- can be added to many verbs, but not all (e.g., roll/unroll; close/*unclose). This study compared theoretical accounts of how this is done. Children aged 5–6 (*N* = 18), 9–10 (*N* = 18), and adults (*N* = 18) rated the acceptability of *un*- prefixed forms of 48 verbs (and, as a control, bare forms). Across verbs, a negative correlation was observed between the acceptability of ungrammatical *un*- prefixed forms (e.g., *unclose) and the frequency of (a) the bare form and (b) alternative forms (e.g., *open*), supporting the *entrenchment* and *pre-emption* hypotheses, respectively. Independent ratings of the extent to which verbs instantiate the semantic properties characteristic of a hypothesized semantic cryptotype for *un*- prefixation were a significant positive predictor of acceptability, for all age groups. The relative importance of each factor differed for attested and unattested *un*- forms and also varied with age. The findings are interpreted in the context of a new hybrid account designed to incorporate the three factors of entrenchment, pre-emption, and verb semantics.

A key question in cognitive science is that of how children acquire their native language. Since the defining characteristic of language is its productivity, one of the most important aspects of this acquisition process is the formation of generalizations that allow speakers to produce novel utterances, while avoiding those that fellow speakers would consider unacceptable.

A problem facing learners is that languages often contain partial regularities, which suggest generalizations that are too broad. For example, in English, many verbs may be used in both the intransitive and transitive construction (e.g., *The plate broke; John broke the plate*). However, the generalization that *any* verb that has appeared in the intransitive construction may appear in the transitive construction is too broad, yielding attested overgeneralization errors such as *He giggled me, *She came it over there, and *I'm just gonna fall this on her (Bowerman, 1988). This is not simply a quirk of the transitive construction. Analogous errors are attested for datives (e.g., Don't say that to me; *Don't say me that), locatives (e.g., She filled the cup with juice; *She filled juice into the cup), and—the focus of this study—reversative *un*- prefixation (all from Bowerman, 1988)^1^:

How do you *unsqueeze it? (wanting clip earring removed)

Mother: I have to capture you (grabbing child). Child: *Uncapture me!

I hate you! And I'm never going to *unhate you or nothing!

He tippitoed to the graveyard and *unburied her (telling ghost story).

I'm gonna *unhang it (taking down stocking from fireplace)

Further *un*- prefixation errors attested in naturalistic and experimental contexts include *unpull, *unflow, *undisappear, *unbuild, *unblow, *unlight, *unknit, and *unstraighten (Bowerman, 1988; Clark, Carpenter, & Deutsch, 1995).

Overgeneralization errors of this type present an intriguing conundrum: Children clearly set up some kind of generalization that allows them to produce *un*- forms that they have not heard previously, or else such errors would not occur. Yet somehow, children become adult speakers who regard at least some unattested *un*- forms as ungrammatical. Note that this problem—and each of the proposed solutions to be investigated in this article—is not specific to *un*- prefixation but applies to all constructions for which overgeneralization errors are observed (including the transitive causative, dative and locative constructions). Thus, while, on the face of it, *un*- prefixation errors may seem to be a somewhat esoteric concern, this domain constitutes a test-case for accounts of the formation and restriction of linguistic generalizations in general; an issue that lies at the very heart of language acquisition research.

The apparent paradox outlined above is sometimes termed the “no-negative-evidence” problem (e.g., Bowerman, 1988; Marcus, 1993; Pinker, 1989), due to the claim that children receive very little feedback from adults regarding the ungrammaticality of particular utterances. However, this would seem to be something of a misnomer, given the growing body of evidence that children not only receive adult feedback but selectively modify their utterances in response to it (Chouinard & Clark, 2003; Clark & Bernicot, 2008; Demetras, Post, & Snow, 1986; Farrar, 1992; MacKey & Philp, 1998; Moerk, 1990; Penner, 1987; Saxton, Backley, & Gallaway, 2005; Strapp, Bleakney, Helmick, & Tonkovich, 2008). For example, in the following exchanges discussed by Chouinard and Clark (2003), the child explicitly accepts a parental recast, in the second case additionally rejecting a recast that does not capture his intended meaning.

Abe (2;5.14): my momma cry.

Father: mommy cried.

Abe: uh-huh you yelling. [Kuczaj, Abe 5:1]

Abe: (2;5.7) the plant didn't cried.

Father: the plant cried?

Abe: no.

Father: oh. the plant didn't cry

Abe: uh-huh. [Kuczaj, Abe 3:163]

Thus, relatively direct negative evidence from caregivers, far from being absent, likely plays an important role in children's learning.

Nevertheless, it would seem unlikely that this type of evidence alone is sufficient to account for all the phenomena observed with regard to the avoidance of overgeneralization error. A number of studies have found that adults reject as ungrammatical errors with very low frequency verbs (e.g., *The funny clown chuckled Lisa; *Homer propelled Bart the ball), for which they are unlikely to have received corrective recasts (Ambridge, Pine, Rowland, & Chang, 2012; Ambridge, Pine, Rowland, Jones, & Clark, 2009). Furthermore, when adults encounter novel verbs, either in an experimental scenario or because a new lexical item has entered the language, they reject certain uses as ungrammatical, without having to first produce an error and receive corrective feedback (e.g., *The funny clown tammed Lisa* [where *tam* is a novel verb meaning to laugh in a particular manner; *The directions texted to John; Ambridge, Pine, & Rowland, 2011). Finally, although recasts occur frequently for common errors (e.g., *hit* for *hitted), some types of overgeneralization error would seem to be too infrequent for sufficient feedback opportunities to occur. For example, a recent dense-database study of the transitive causative construction (Theakston, Maslen, Lieven, & Tomasello, 2012) failed to find a single overgeneralization error of the type observed in Bowerman's (1988) diary study (*He giggled me, *She came it over there and *I'm just gonna fall this on her). Indeed, Bowerman (1988: 92) characterizes *un*- prefixation errors as “one-time errors” for which “learners do not have repeated opportunities to observe the way other people express this particular meaning.” For these infrequent errors, the question is not so much how children retreat from the few errors that they produce, but how they generally avoid such errors, while maintaining the capacity to produce novel grammatical utterances using the same productive generalization. None of these arguments are intended to deny the importance of recasts and other forms of corrective feedback in the restriction of generalizations^2^; the claim is simply that this is unlikely to be the whole story.

Three mechanisms for the retreat from (or avoidance of) overgeneralization errors have been proposed: *pre-emption, entrenchment,* and the formation of *semantic verb classes*^3^ Although all enjoy some empirical support, none is able to account for the phenomenon in its entirety, and many struggle in particular in the domain of *un*- prefixation. This is problematic, as any successful account of the restriction of overgeneralization errors will presumably encompass overgeneralization errors of all types. Thus, the aim of this study is to investigate which of the predicted effects are observed in the domain of *un*- prefixation and, subsequently, whether it is possible to arrive at a hybrid account that yields all the observed effects.

The first of these proposals, pre-emption (Clark, 1993; Clark & Clark, 1979) originates in the domain of word-learning, where the conventional adult form for expressing a particular meaning (e.g., *pyjamas; to row*) gradually comes to pre-empt or block children's own coinages (e.g., *sleepers; *to oar). Braine and Brooks (1995) and Goldberg (1995) extended the notion of pre-emption to cover argument structure overgeneralization errors. For example, periphrastic causative uses of *fall* (e.g., *I'm gonna make this fall on her*) are hypothesized to block transitive causative overgeneralizations (e.g., *I'm gonna fall this on her). It is currently unclear whether pre-emption can operate for all the different types of error that children produce. Pre-emption works best when the adult form and the child coinage are perfectly synonymous. Hence, it is almost certainly the sole or predominant mechanism by which children retreat from past-tense over-regularization errors (e.g., *hit* and *sat* pre-empt the common child forms *hitted and *sitted, respectively). Boyd and Goldberg (2011) also found that pre-emption offers the best account of how speakers avoid errors with *a*-adjectives (e.g., *the asleep boy), which are blocked by relative clause uses (e.g., *the boy that's asleep*).

Extending the notion to argument structure overgeneralizations (e.g., *I'm gonna make this fall* for *I'm gonna fall this) is described by Bowerman (1988: 91) as “a stretch” because the attested and hypothesized forms are not perfect synonyms: “lexical causatives and their periphrastic counterparts differ with respect to the directness and conventionality of the act of causation specified (compare, for example, *John stood the baby up* [direct physical causation] and *John made the baby stand up* [indirect causation, e.g., through giving an order]).” Nevertheless, there is some evidence for pre-emption both for this construction (Brooks & Tomasello, 1999; Brooks & Zizak, 2002; though see Ambridge & Lieven, 2011: for a caveat; Ambridge et al., 2011) and the dative constructions (Ambridge, Pine, Rowland, & Freudenthal, in press). As predicted by this account, the greater the frequency of a potentially pre-empting form, the less likely children are to produce overgeneralization errors, or to rate them as acceptable in a judgment task.

Can pre-emption be extended to *un*- prefixation errors? For Bowerman (1988: 91), this is a stretch too far:

Here, the child meets with no consistent alternatives in the adult input, For instance, in contexts where *unsqueeze* would be appropriate, if it existed, adults might say *loosen, ease up, release, let go, remove,* and so on. None of these is in direct semantic competition with *unsqueeze*, since none of them specifies or requires that the event referred to is the reversal of an act of “squeezing.” Nor should the child take the existence of such forms as having any bearing on the possibility of *unsqueeze*: reversative *un*- forms coexist harmoniously with various related constructions, for example, *unwrap* and *take the wrapper off, unzip* and *pull the zipper down, unload* and *empty*…A learner cannot take every sentence he hears as precluding all sentences that express somewhat related messages; natural languages are too rich for this.

Nevertheless, it would seem premature to dismiss a priori the possibility that pre-emption can operate in the domain of *un*- prefixation. It could be, for example, that children do allow *take the wrapper off* to block *unwrap* until such time as they hear the latter form attested in the input. In this study, we investigate empirically whether a pre-emption account can be applied to the domain of *un*- prefixation by asking adults to suggest pre-empting alternatives for ungrammatical *un*- forms. The pre-emption account predicts a negative relationship between the acceptability of an over-general *un*- form and the availability of pre-empting alternatives. As noted by an anonymous reviewer, if any pre-emption effect is observed, we would not necessarily expect to see it in very young children. Even for simple cases involving past-tense errors (e.g., *sat* for *sitted), the over-general form and the competing adult alternative co-exist for a long period before the former is abandoned (Maslen, Theakston, Lieven, & Tomasello, 2004; Ramscar & Yarlett, 2007). Thus, it may be that any effect of pre-emption is not visible until relatively late in childhood.

The entrenchment hypothesis (Braine & Brooks, 1995) states that repeated presentation of a verb leads to an ever-increasing probabilistic inference that non-attested uses (e.g., *I'm gonna fall this on her) are not permitted. This account is similar in many respects to the pre-emption hypothesis, but with one important difference. Under the pre-emption hypothesis, an over-general form (e.g., *I'm gonna fall this on her) is probabilistically blocked by only nearly synonymous forms (e.g., *I'm gonna make this fall on her*). Under the entrenchment hypothesis, such an error is probabilistically blocked by *any* use of the relevant item (e.g., *I'm gonna make this fall on her; It fell on her; Will it fall on her?* etc.). Intuitively, the inference is that if a particular item has been encountered many times but never in a particular construction, this absence must reflect ungrammaticality rather than mere coincidence (Hahn & Oaksford, 2008). This inference from absence is demonstrated more formally in Bayesian rational-learner mathematical models of the retreat from overgeneralization (e.g., Alishahi & Stevenson, 2008; Chater & Vitanyi, 2007; Dowman, 2000; Hsu, 2009; Onnis, Roberts, & Chater, 2002; Perfors, Tenenbaum, & Wonnacott, 2010; Perfors et al., 2011).^4^ Experimental studies have shown that, as predicted, both the rated acceptability and production probability of overgeneralization errors decreases with increasing verb frequency (e.g., Ambridge et al., 2009, 2011, 2012, in press; Brooks, Tomasello, Dodson, & Lewis, 1999; Stefanowitsch, 2008; Theakston, 2004; Wonnacott, Newport, & Tanenhaus, 2008).

Whether entrenchment can also account for the retreat from error in the domain of *un*- prefixation remains unclear. Entrenchment works well when both the relevant individual verbs and the target construction are reasonably frequent in the input, thus allowing the learner to rapidly build the inference that the two co-occur less often than would be expected, given the frequency of the verb and target construction independently. For example, even young children rate *The joke laughed the man as unacceptable, since both the verb *laugh* and the transitive construction are relatively frequent. In contrast, many of the relevant verbs, and the *un*- prefixation construction itself, are relatively rare. Furthermore, some verbs are considerably more frequent in bare form than in the *un*-[VERB] construction (e.g., *twist*), while others (e.g., *cork, leash*) show the opposite pattern. This is particularly problematic for versions of the account under which learners must calculate the expected co-occurence of the verb and the *un*- prefixation construction given their independent frequencies (e.g., Stefanowitsch, 2008). Even for simpler formulations based solely on verb frequency, the relative scarcity of many of the relevant verbs means that entrenchment will presumably remain unreliable until a very large volume of data has been encountered.

This study tests the prediction of the entrenchment hypothesis of a negative correlation between the rated acceptability of *un*- prefixation errors (e.g., *unsqueeze, *unfill) and the overall frequency of the relevant verb (e.g., *squeeze, fill*, in all forms except with the prefix *un*-) in a representative corpus. The methodology of this study also allows for direct comparison of the pre-emption and entrenchment hypotheses. The former predicts that the best predictor of the relative unacceptability of a particular error (e.g., *unsqueeze vs. *unfill) is some composite measure of the frequency of possible competing forms (e.g., *release* + *loosen* vs. *empty* + *drain*). The latter predicts that the best predictor of the relative unacceptability of a particular error (e.g., *unsqueeze vs. *unfill) will be the overall frequency of the relevant “bare” rorms (e.g., *squeez*[*es/ed/ing*] vs. *fill*[*s/ed/ing*]).

The third previous account of the retreat from overgeneralization is Pinker's (1989) *semantic verb class hypothesis*. Under this account, learners form classes of semantically similar verbs that are restricted to particular constructions. For example, verbs denoting “semi-voluntary expression of emotion” (Pinker, 1989: 303) may appear in the intransitive construction (e.g., *Lisa giggled/laughed/chuckled/sniggered*) but not the transitive (e.g., *The funny clown giggled/laughed/chuckled/sniggered Lisa), while “manner of motion” verbs may appear in both (e.g., *The ball bounced/rolled/slid; Lisa bounced/rolled/slid the ball*). The classes are not arbitrary but relate to the “semantic core” of each construction. For example, the transitive causative construction is claimed to be associated with verbs of direct external physical causation, with which internally caused single-participant events of the *laughing* type are incompatible. Classes of the *bounce/roll* type alternate between the intransitive and transitive construction, as they denote events that are mid way between internal and external causation. For example, an item must have certain properties to *bounce* or *roll* and can do so with no external cause other than gravity. On the other hand, these events are amenable to direct external physical causation in a way that verbs from transitive-only classes (e.g., *laugh, speak, swim*) are not. Pinker's (1989) account explains the findings of Ambridge et al. (2008, 2011), that learners appear to respect these semantic classes, when taught novel verbs consistent with particular classes (see also Brooks & Tomasello, 1999, for production).

The proposal that learners use verb semantics to restrict verb generalization has proved controversial. A sceptical position is that while particular restrictions might ultimately have a semantic motivation (or have had one historically), actual learners do not need to be aware of this motivation, and learn verbs' restrictions via solely pre-emption/entrenchment (e.g., Stefanowitsch, 2008). Probably most widely held is the position that while learners use semantics to restrict inappropriate generalization of some verbs, others have entirely idiosyncratic properties that can be learned only distributionally, via pre-emption/entrenchment (e.g., Bowerman, 1988; Boyd & Goldberg, 2011; Braine & Brooks, 1995). A related position is that learners start out using pre-emption/entrenchment, and it is only after they have acquired some verb restrictions on this basis that they become aware of correlations with verb semantics that can be used to restrict subsequent generalization (e.g., Tomasello, 2003; see also Wonnacott et al., 2008; Wonnacott, 2011; Perfors et al., 2010, 2011). In contrast, although he does not discuss pre-emption or entrenchment, Pinker (1989:103) is explicit in his aim to “leave no negative exceptions” to his semantics-based account.^5^

Applying this proposal to the domain of *un*- prefixation is not straightforward (Pinker, 1989; does not attempt to do so). The semantic verb class hypothesis works well when the relevant verbs cluster tightly into semantically based classes such as verbs of “contained motion taking place in a particular manner (e.g., *slide, skid, float, roll, bounce*)” (Pinker, 1989: 1303), which may appear in both the intransitive and transitive construction (e.g., *The ball rolled; John rolled the ball*). However, this would not seem to be the case for *un*- prefixation, where the author is aware of no proposals for discrete semantically based classes of verbs that do and do not appear in the construction. Indeed, it may well be the case that such a classification is not possible. Whorf (1956) argued that verbs that may take *un*- constitute a “semantic cryptotype” (or “hidden rule”). Although his use of the term is somewhat inconsistent, the notion that Whorf seems to have in mind here is that of a fuzzy, probabilistic family resemblance category. Although, as a group, verbs that may take the reversative *un*- prefix share certain meaning components (e.g., *covering, enclosing, surface-attachment, circular motion, hand-movements*, *change of state*), no individual property would seem to be either necessary or sufficient for membership.^6^

In addition to the entrenchment and pre-emption hypotheses, this study tests Whorf's (1956) proposal that learners are forming a probabilistic, semantically based category (or “cryptotype”) of verbs that take the reversative prefix *un*-. This requires some quantitative measure of the extent to which individual verbs are consistent with the cryptotype. The measure chosen was adult ratings of the extent to which each of 48 verbs exhibits each of 15 semantic properties proposed by Whorf (1956) as relevant to the cryptotype (taken from a previous computational-modeling study). If learners are indeed forming a cryptotype of this nature, then these ratings should predict the relative acceptability of the *un*- prefixed form across verbs—as rated by adults and children—even after controlling for the following factors (outlined in more detail in the Methods section):

1. *Existence of the un- form*: One possible strategy is simply to rate all *un*- forms that have been previously encountered as highly acceptable and all *un*- forms that have not been previously encountered as highly unacceptable. It is therefore important to control for whether each verb is attested in *un*- form in a representative corpus.
2. *Frequency of the un- form*: Looking within attested *un*- forms, it seems likely that more frequently occurring forms will be rated as more acceptable. It is therefore important to control not only for attestedness of each *un*- form but also for its frequency.
3. *Acceptability of the bare form:* A common finding in acceptability judgment studies is that participants simply like some verbs (e.g., those that are familiar, phonologically prototypical and so on) more than others. Only after controlling for the acceptability of individual verb per se is it possible to investigate their relative acceptability in *un*- form.
4. *Reversibility:* A potential objection is that there is no learnability paradox in this domain, as only verbs that denote reversible actions may be prefixed with *un*-. Although this is not obviously the case (e.g., one could, in principle, *unclose a door or *unlift one's arms), we control for this possibility by obtaining reversibility ratings for all verbs.
5. *Pre-emption:* This is a composite measure of the frequency of potentially pre-empting forms (e.g., *release* + *loosen* for *unsqueeze) supplied by the reversibility raters in a second task.
6. *Entrenchment:* This measure is simply the total frequency of the relevant bare form (e.g., *squeez*[*es/ed/ing*] for *unsqueeze).

If, having controlled for all these factors, ratings of the extent to which individual verbs exhibit the semantic properties posited for the *un*- cryptotype significantly predict the acceptability of individual *un*- forms, this would constitute evidence for the psychological reality of some kind of semantically based generalization. An additional possibility is that the relative acceptability of individual *un*- forms may be wholly or partly predicted by the frequency of competing forms that express a similar meaning (pre-emption) or of the bare (i.e., non *un*- prefixed) form of the verb.

This study tested these predictions by obtaining from children (aged 5–6 and 9–10 years) and adults acceptability ratings for the *un*- form (and—as a control—the bare form) of 48 verbs. Three age-groups were studied to investigate the developmental trajectory of the three proposed mechanisms, and whether their relative influence changes with age.^7^

## 1. Method

### 1.1. Participants

Participants for the main part of the study (grammaticality judgments) were 18 children aged 5;0–6;0 (*M =* 5;6), 18 children aged 9;10–10;10 (*M =* 10;5), and 18 adults aged 18–21, with an equal number of males and females at each age. All were normally developing monolingual speakers of British English and were primarily from a middle-class background (though detailed SES information was not collected). Children were recruited via—and tested at—their school in the North West of England; parents were sent a letter outlining the study, and a consent form. The adults were first-year undergraduate psychology students who completed the study as part of their course requirements. None had studied child language acquisition or linguistics. A second group of 15 adults were recruited from the same population to serve as raters of reversibility and to provide potential pre-empting forms (see below).

### 1.2. Materials and procedure

Forty-eight verbs were selected from the study of Li and MacWhinney (1996), a study in which a connectionist model was trained to classify verbs as either taking or not taking *un*-. According to Li and MacWhinney's (1996) original classification, 24 of these verbs are prefixable with *un*- (“*un*- verbs”), while 24 are not (“zero verbs”). However, because these classifications seemed somewhat doubtful (and perhaps more representative of American than British English), the verbs were reclassified as according to whether the *un*- form appears in the *British National Corpus* (BNC), a 100 million word corpus of spoken and written British English. The resulting classifications were as follows.

*un*- verbs (*N* = 31): *Bandage, Believe, Bend, Buckle, Button, Chain, Cork, Crumple, Delete, Do, Embarrass, Fasten, Freeze, Hook, Lace, Latch, Leash, Lock, Loosen, Mask, Open, Pack, Reel, Roll, Screw, Snap, Tie, Tighten, Veil, Wrap, Zip*.

zero- verbs (*N* = 17): *Allow, Ask, Close, Come, Fill, Give, Go, Lift, Press, Pull, Put, Release, Remove, Sit, Squeeze, Stand, Straighten*.

Although differences of opinion are perhaps inevitable, these classifications have the benefit of being objective and based on actual usage (as opposed, for example, to a dictionary). Indeed, as we shall see shortly, they are an excellent predictor of the extent to which speakers rate the relevant *un*- forms as acceptable. That said, it is important to bear in mind that the binary classification of verbs as *un*-/zero is somewhat artificial, given that many verbs classified as taking *un*- had very low *un*- - form frequency in the BNC (e.g., *unbandage, uncrumple, undelete, unbelieve, unloosen,* and *untighten* were each attested just once), and so may be less than fully acceptable, at least for some speakers.

**Fig. 1:**
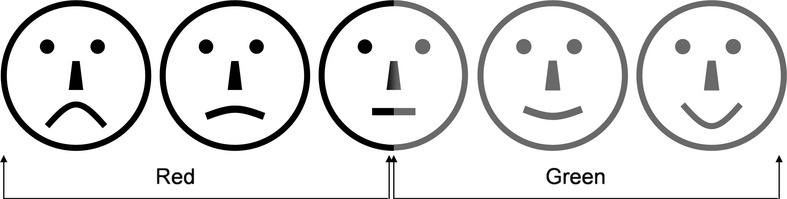
The five-point smiley face scale used by participants to rate the relative acceptability of the un- prefixed and bare verb forms (reproduced from Ambridge et al., 2008: 105, by permission of Elsevier).

For each verb, participants rated the acceptability of both the *un*- form (e.g., *unsqueeze) and the bare form (e.g., *squeeze*), using a five-point smiley face scale (see Fig. 1). Details of this procedure can be found in Ambridge et al. (2008) and Ambridge (2011). In brief, participants select a green or red counter to indicate whether the form is acceptable or unacceptable, then place the counter on the scale to indicate the degree of (un)acceptability. Nine training sentences containing correct past-tense forms and overgeneralization errors (e.g., Lisa *eated the ice cream) were used to train participants in the correct use of the scale.^8^ To make the study more engaging, and to ensure correct interpretation, for all training and test trials, the verb was presented in a sentence (e.g., Bart *unclosed the box) and illustrated with a “before and after” picture, presented on a laptop computer. During both the training and test trials, it was emphasized to participants that the task was to rate the acceptability of the verb form, not the entire sentence. As an additional precaution, two counterbalance sets with a different sentence for each verb were created. As ratings for the two sets were not significantly different (*t* < 1, *p* = n.s.), and were highly correlated (*r* = .84, *p <* .001), all analyses were collapsed across both sets. The full set of training and test sentences can be found in the Appendix.

Because it was felt that 96 trials would be too arduous for children, the items were divided into three sets of 32 sentences (one *un*- form and one bare form for each verb), with each child rating only one set. To control for set, verb was included as a random effect in all statistical analyses. Participants completed the test trials in random order with the constraint that the *un*- and bare form of a given verb were never presented in consecutive trials.

### 1.3. Predictor variables

To test each of the individual predictions outlined above while controlling for potentially confounding variables, linear mixed-effects regression models were fitted to the data. The outcome variable was the acceptability rating for each *un*- form on the five-point scale. Because the data are bounded (ratings cannot be lower than 1 or >5) and not normally distributed, they were subjected to a log-odds transformation. This effectively treats the five different points on the rating scale as different categories, though categories that line up in a particular order. The predictor variables are listed below. The first four are control predictors designed to rule out theoretically uninteresting effects and task-strategies; the final three test the accounts under investigation. As detailed below, it was often necessarily to residualize particular predictors against others, sometimes due to observed co-linearity, sometimes for theoretical reasons (i.e., because it is desirable in principle to control for the frequency of each attested *un*- form, regardless of whether this predictor is in practice correlated with others).

#### 1.3.1. Existence of un- form (verb type)

Verb type (*un/zero*, as outlined above) was entered into the regression model as a categorical predictor variable (1/0) to control for the fact that participants will have heard many of the verbs in *un*- form.

#### 1.3.2. Frequency of the un- form

For verbs that exist in *un*- form, participants are likely to give higher acceptability ratings to more frequently encountered forms. To control for this possibility, frequency of each *un*- form in the BNC (both spoken and written) was included as a predictor variable. It was necessary to use the entire BNC, as opposed to the spoken subsection (approximately 10% of the corpus) or a corpus of child-directed speech, to ensure that the frequency counts are reliable. Many perfectly acceptable *un*- forms are relatively infrequent, and so do not appear in smaller corpora. Counts were natural log (*N* + 1) transformed, then residualized against verb type. This ensures that the frequency measure captures variance in the frequency of attested *un*- forms only. In other words, the fact that some verbs are unattested in *un*- form is captured by the predictor of verb type, not *un*- form frequency. Note that if speakers do not form a productive generalization, and simply base their acceptability ratings on whether—and how often—they have encountered particular *un*- forms, these two predictors would capture the vast majority of the variance in judgments, with subsequent predictors adding little or nothing.

#### 1.3.3. Acceptability of the bare form

For each *un*- form (e.g., *unsqueeze), participants also rated the acceptability of the equivalent bare form (e.g., *squeeze*). These ratings were included as a control predictor, to factor out any effects arising from the fact that participants typically show a general preference for some verbs (perhaps based on familiarity, phonological typicality, social desirability of the event described, and so on). Conversely, these ratings also control for any possible trade-off effect resulting from the rating task. Although there is no particular reason to expect this pattern, it is hypothetically possible that participants may show a dispreference for *un*- forms of verbs that they deem highly acceptable in bare form.

#### 1.3.4. Reversibility

An anonymous reviewer (and members of several conference audiences) have suggested that there is no paradox here: The *un*- prefix may be applied only to verbs that denote reversible actions. Although this is not obviously the case (as noted above, one could, in principle, *unclose a door or *unlift one's arms), basing *un*- form ratings on reversibility is certainly a plausible task strategy. To control for this possibility, reversibility ratings for each verb were obtained from 15 adult participants. Importantly, verbs were presented in bare (stem) form only, and participants were not informed that the study was related to *un*- prefixation. The instructions to participants were as follows:

Some actions are reversible. For example, if a shopkeeper raises his prices, he can reverse this action by lowering them. Some actions are not reversible. For example, if a chef bakes a cake, he cannot reverse this action to end up with the raw ingredients. Some actions are somewhere in between. For example, if a chef boils his soup, he can reverse this action by cooling it down again, but the reversal will not be quite complete as the flavor and texture of the soup will have changed. This study comprises a list of 48 actions. For each action, your task is to rate the extent to which the action is or is not reversible on a seven-point scale).

For each verb, a mean reversibility rating was calculated by taking the mean across participants. Because, like the grammaticality judgments, these ratings are bounded and non-normal, they were also subjected to a log-odds transformation. As for the frequency measure, reversibility was residualized against verb type to control for the fact that only some verbs are attested in *un*- form.

#### 1.3.5. Pre-emption measure

Subsequently, the same group of 15 adult participants were subsequently asked, for each verb, to

think up as many words as you can (maximum = 5) that mean the reversal of this action. Sometimes, there may be no suitable word, but always try as hard as you can to come up with at least one, even if it is not precisely the right meaning. However, you should NOT write words that you would consider “ungrammatical” (i.e., not real English words). VERY IMPORTANT: You MAY NEVER write an *un*- word, even if this word has the right meaning. For example, if the action is *bolt*, then *unbolt* would have the right meaning BUT YOU MAY NOT WRITE UNBOLT.^9^ Instead, you must try to come up with alternatives that do NOT start with *un*-.

Because it was necessary to mention *un*- prefixation, the order of the reversibility and pre-emption tasks was not counterbalanced. This was to avoid the possibility that participants could use the availability or otherwise of an *un*- form, or pre-empting alternatives, to judge reversibility. For each verb, the two most commonly suggested pre-empting forms (e.g., for *unsqueeze, *release* and *loosen*) were selected, and their (natural log *N* + 1) frequencies obtained from the BNC and summed to yield the pre-emption measure. Various other measures were calculated (e.g., frequency of the single most commonly suggested form, sum frequency of the top two forms/all forms, number of suggested forms, number of participants suggesting the top form), but all were less successful as predictors of *un*- form acceptability. Presumably, the advantage of including only the two most commonly suggest forms—as opposed to all forms—is that it excludes the more marginal examples that were only suggested by one or two participants. To control for the any negative correlation between the existence/frequency of an *un*- form and the frequency of pre-empting alternatives, the pre-emption measure was residualized against verb type and *un*- form frequency.

#### 1.3.6. Entrenchment measure

The entrenchment measure is simply the (natural log *N* + 1) frequency of the bare (i.e., without *un*-) form of the relevant verb in the BNC (all texts). All inflected forms (e.g., *squeeze, squeezes, squeezed, squeezing*) were counted, regardless of sentence context; *un*- prefixed forms were excluded (in fact, they were never included, as counts used the BNC lemmatization system, which treats morphologically derived forms as separate words). The entrenchment measure was not residualized against *un*- form frequency, acceptability of the bare form, reversibility or the pre-emption measure, as there is no theoretical reason to expect any colinearity with these measures (and, as we shall see shortly, none was observed). The entrenchment measure was, however, residualized against verb type (*un*-, zero), to allow for investigation of the prediction that—even taking into account the status of the *un*- form as attested or unattested—frequency of the bare form will predict the degree of ungrammaticality. This also controls for an unintentional confound in the stimulus set, whereby the *un*- verbs are of lower frequency in bare form than the zero verbs (with mean BNC counts of around 20,000 and 46,000, respectively).

#### 1.3.7. Semantic cryptotype measure

To test the claim that speakers use verb semantics to help determine whether particular verbs may appear with *un*-, semantic feature ratings were obtained from the study of Li and MacWhinney (1996). These authors asked 15 native English speakers to rate each verb as to whether it instantiates each of 20 semantic features:

(1) Mental activity, (2) Manipulative action, (3) Circular movement, (4) Change of location, (5) Change of state, (6) Resultative, (7) A affects B, (8) A touches B, (9) A distorts B, (10) A contains B, (11) A hinders B,(12) A obscures B, (13) A surrounds B, (14) A tightly fits into B, (15) A is a salient part of B, (16) A and B are separable, (17) A and B are connectable, (18) A and B are interrelated, (19) A and B are in orderly structure, (20) A and B form a collection.

These semantic features were chosen specifically as being relevant to determining the possibility of *un*- prefixation and were based mainly on Whorf (1956). This is because the claim is that *un*- prefixability is based not upon very general semantic features (e.g., ACT, HAVE, GO), but on a cluster of narrow-range semantic properties that are specifically relevant to this domain. Thus, each verb had a score of between 0 and 15 for each semantic feature, corresponding to the number of participants who judged the feature to be relevant to the verb's meaning. Because running a regression analysis with 20 semantic predictors and only 48 data points is statistically problematic, Principal Components Analysis was used to condense these ratings, selecting the single component with the largest Eigenvalue (6.37). A further five components were disregarded as all had Eigenvalues in the range 1–2 and explained very little additional variance. The outcome is a semantic feature score, which represents the extent to which each verb exhibits the cluster of semantic features thought to be relevant for *un*- prefixation. The features with large (>0.5) loadings on this factor were (in decreasing order of magnitude): Change of location (0.92), A and B are separable (0.91), A touches B (0.78), Mental activity (0.72), A and B are interrelated (0.71), A hinders B (0.69), Circular movement (0.68), A is a salient part of B (0.63), and A and B form a collection (−0.51). The fact that the analysis identified nine features associated with broadly similar loadings on this factor (as opposed—for example—to a single feature with a very large loading) provides some support for Whorf's notion of a complex semantic cryptotype.

This semantic feature score was residualized against verb type, to allow for investigation of whether semantics predict not only which verbs do and do not take *un*-, but the relative acceptability of particular *un*- forms. Although none of these features relate straightforwardly to reversibility per se, the semantic feature score was also residualized against this factor, to address the potential objection that these features are somehow a proxy for reversibility. The semantic feature score was also residualized against bare-form frequency since post-hoc inspection revealed that that the two were highly correlated (*r* = .5, *p* < .01). Why this should be is unclear, but one possibility is that participants are more reluctant to attribute semantic features to low-frequency verbs for which they are unsure of the precise meaning.

Finally, it is important to make explicit the fact that while grammaticality judgments were obtained from both adults and children, semantic ratings (as well as the reversibility and pre-emption measures) were obtained from adults only. There are two reasons for this: one methodological, one theoretical. The methodological reason is that it was considered unlikely that children—particularly the youngest—would be able to provide meaningful ratings of these rather abstract semantic features. The theoretical reason is that it is the *adult* conception of a verb's meaning that is held to determine the extent to which it is compatible with *un*- prefixation. Thus, the use of adult semantic ratings allows for investigation of the prediction that children's errors are caused by non-adultlike knowledge of verb semantics. If this is the case, then the proportion of variance explained by this predictor should increase with development, as children's knowledge of semantics reaches the adult state.

## 2. Results

**Fig. 2:**
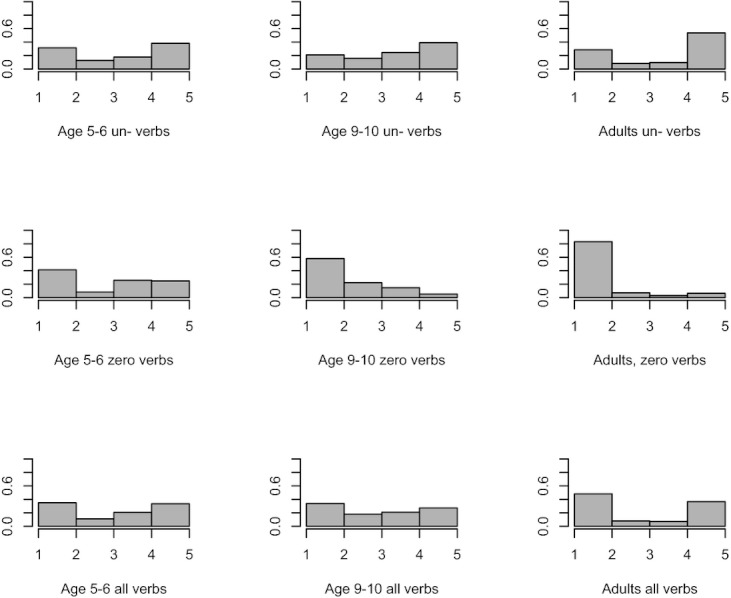
Distribution of ratings of un- forms for ages 5–6, 9–10, and adults for (first row) verbs that may take un (un- verbs), (second row) verbs that may not take un- (zero verbs), and (third row) all verbs combined.

**Fig. 3:**
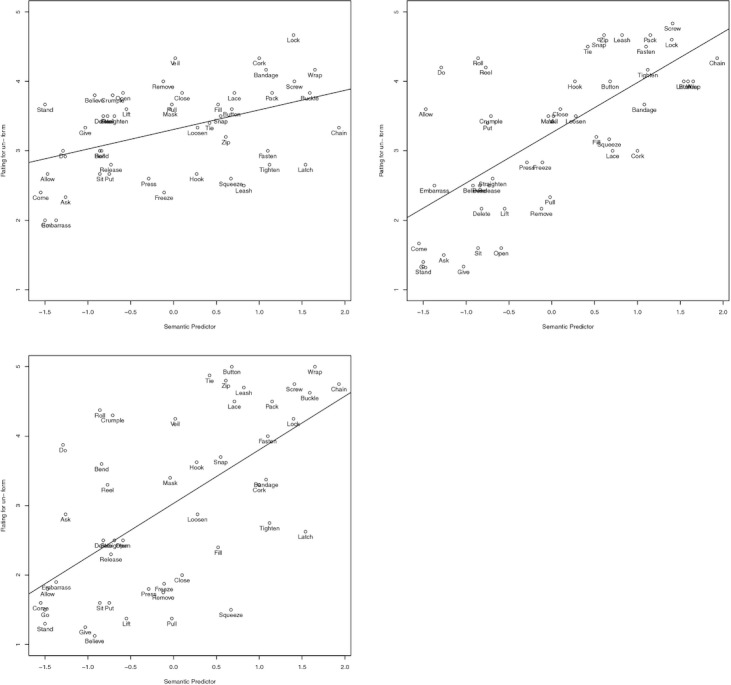
Mean acceptability rating for the un- form of each verb by age group (5–6, 9–10, adults) as a function of the semantic-features predictor.

Figure 2 illustrates the distribution of ratings for *un*- forms of verbs that (a) can (*un*- verbs) and (b) cannot (zero verbs) be grammatically prefixed with *un*- (i.e., for verbs that do/do not occur with this prefix in the BNC). This figure also shows the distribution of *un*- form ratings for (c) all verbs combined. It remains clear that adults show the greatest tendency towards binary performance (i.e., rating each *un*- form as either 5 or 1), and youngest children the least. It is also clear that the data are not normally distributed, hence necessitating the log-odds transformation described earlier. Figure 3 displays the mean untransformed ratings for the *un*- prefixed form of each verb, plotted against the semantic predictor. Note that because this figure displays the raw semantic predictor (i.e., with other factors not partialled out) and the raw untransformed ratings, it does not correspond directly to the statistical analyses described below; its purpose is simply to allow for an intuitive understanding of the relationship between the semantic feature measure and participants' acceptability ratings. It will be observed that, for all ages, both the semantic and acceptability ratings are distributed relatively evenly across their respective scales and that, as predicted, there is a reasonably large positive correlation between the two.

The data—participants' ratings of *un*- forms—were analyzed in the R environment using linear mixed-effects regression models (*lmer* from the package *lme4*; Bates, Maechler, & Bolker, 2011) with *p*-values estimated using *pvals.fnc* from the package *languageR* (Baayen, 2011). Each of the predictor variables listed in the previous section was included as a fixed effect. Participant and item (verb) were included as random effects. In addition to the residulization procedures outlined above, all predictor variables were centered to minimize the effects of multicollinearity. The correlations between predictor variables are shown in Table 1. The precautions taken to reduce multicollinearity appear to have been largely successful, with the majority of correlations smaller in magnitude than *r* = .05, and the largest *r* = .22.

**Table 1:** Correlations between the predictor variables

	Verb Type	Freq Un- Form	Rating for Bare Form	Reversibility	Freq Pre-Empting Forms	Freq Bare Form (ent)
Age 5–6						
Freq un- form	−0.01					
Rating for bare form	0.01	0.03				
Reversibility	0.00	−0.14	0.00			
Freq pre-empting forms	−0.01	0.03	0.05	0.02		
Freq bare form (ent)	−0.03	−0.03	−0.16	−0.17	−0.07	
Semantics—factor 1	−0.03	−0.15	−0.03	0.02	−0.11	0.03
Age 9–10						
Freq un- form	0.02					
Rating for bare form	0.22	0.07				
Reversibility	−0.01	−0.14	0.02			
Freq pre-empting forms	0.01	0.02	0.01	0.01		
Freq bare form (ent)	−0.07	−0.02	−0.19	−0.18	−0.05	
Semantics—factor 1	−0.04	−0.15	−0.10	0.02	−0.10	0.04
Adults						
Freq un- form	0.03					
Rating for bare form	0.17	0.16				
Reversibility	−0.01	−0.15	−0.05			
Freq pre-empting forms	0.01	0.02	0.08	0.00		
Freq bare form (ent)	−0.02	−0.01	−0.08	−0.19	−0.07	
Semantics—factor 1	−0.01	−0.16	−0.09	0.04	−0.11	0.02

Following the suggestion of a reviewer, development was investigated by running a separate analysis for each age group and comparing the results, as opposed to running a single analysis including age and its interactions (which leads to an unacceptably high ratio of predictors to data points). However, it is important to note that if such an analysis is run, adding age and its interactions significantly improves the coverage of the model by log-likelihood test, with many of the individual interaction terms significant.

Three analyses were conducted for each age group. The first consisted of a single model including all seven predictors (four control predictors plus the pre-emption, entrenchment and verb-semantics measures). Because every predictor functions as a control predictor for at least one other, non-significant predictors were not removed. For example, while the entrenchment measure is not a significant predictor for the youngest group (*p* = .13), it still explains some (non-significant) portion of unique variance that must be controlled for when investigating any effect of the semantic predictor.

For the second and third analyses, the first analysis was repeated separately for verbs that (a) do not and (b) do appear with the prefix *un*- in the BNC (i.e., zero verbs [*N* = 17] and *un*- verbs [*N* = 31], respectively). These analyses are necessary because the key predictions of the pre-emption and entrenchment hypothesis—increasing unacceptability with increasing frequency of pre-empting/entrenching forms—relate only to ungrammatical forms, and hence only to zero verbs. These analyses also allow for investigation of whether the semantic predictor accounts for variance in both (a) the unacceptability of ungrammatical *un*- forms and (b) the acceptability of grammatical *un*- forms, or simply differentiates the two classes of verbs (i.e., those that may not/may undergo prefixation with *un*-). That said, it is important to bear in mind that the binary classification of verbs as *un*-/zero is somewhat artificial, given the existence of some very low frequency un- forms (e.g., *unbandage, uncrumple, undelete*) that may not be fully grammatical for all speakers.

The results of these analyses are shown in Table 2 (all verbs), Table 3 (zero verbs), and Table 4 (*un*- verbs). Note that while the numerical values are in units of log-odds-transformed ratings, and are hence not readily interpretable, the sign of the beta values is informative: A positive value indicates that increasing the value of the predictor resulted in increased acceptability of the *un*- form, as would be predicted for verb type (coded as 0/1 for zero/*un*), frequency of the *un*- form, reversibility, semantic features, and—perhaps—acceptability of the bare form (assuming an effect of general verb preference rather than a trade-off effect). A positive value indicates that increasing the value of the predictor resulted in decreased acceptability of the *un*- form, as would be predicted for the pre-emption and entrenchment measures.

**Table 2:** Mixed-effects models for all verbs combined (*N* = 48)

	Age 5–6						Age 9–10						Adults					
			
				HPD95 CIs						HPD95 CIs						HPD95 CIs		
															
Fixed Effects	*M* (β)	*SE*	*t*	Lower	Upper	*p*	*M* (β)	*SE*	*t*	Lower	Upper	*p*	*M* (β)	*SE*	*t*	Lower	Upper	*p*
(Intercept)	(0.54)	(0.20)	(2.63)	(0.15)	(0.91)	(.01)	−(0.17)	(0.14)	−(1.21)	−(0.47)	(0.07)	(.23)	−(0.64)	(0.14)	−(4.53)	−(0.88)	−(0.37)	(.00)
Verb type	**0.38**	**0.14**	**2.60**	**0.08**	**0.68**	**.01**	**1.31**	**0.14**	**9.04**	**1.05**	**1.57**	**.00**	**1.79**	**0.17**	**10.79**	**1.49**	**2.11**	**.00**
Freq un- form	0.01	0.04	0.23	−0.08	0.10	.82	**0.15**	**0.04**	**3.51**	**0.08**	**0.23**	**.00**	**0.28**	**0.05**	**5.32**	**0.18**	**0.39**	**.00**
Rating for bare form	−0.02	0.06	−0.25	−0.12	0.12	.80	**0.23**	**0.07**	**3.09**	**0.08**	**0.36**	**.00**	**0.31**	**0.07**	**4.33**	**0.16**	**0.45**	**.00**
Reversibility	**0.23**	**0.12**	**1.93**	**−0.02**	**0.46**	**.05**	−0.05	0.11	−0.41	−0.25	0.18	.68	−0.08	0.13	−0.60	−0.33	0.15	.55
Freq pre-empting forms	0.01	0.03	0.38	−0.05	0.07	.71	**−0.07**	**0.03**	**−2.57**	**−0.13**	**−0.02**	**.01**	**0.00**	**0.03**	**0.06**	**−0.06**	**0.06**	**.95**
Freq bare form (ent)	−0.06	0.04	−1.52	−0.14	0.01	.13	**−0.09**	**0.04**	**−2.51**	**−0.16**	**−0.02**	**.01**	**−0.11**	**0.04**	**−2.57**	**−0.19**	**−0.02**	**.01**
Semantics—factor 1	**0.20**	**0.10**	**2.12**	**−0.01**	**0.40**	**.03**	**0.49**	**0.09**	**5.18**	**0.33**	**0.67**	**.00**	**0.29**	**0.11**	**2.55**	**0.09**	**0.51**	**.01**

Random Effects	Var						Var						Var					
Verb	0.00						0.09						0.17					
Participant	0.47						0.10						0.02					
Residual	1.28						0.73						1.12					

**Table 3:** Mixed-effects models for zero-verbs (verbs that do NOT take un-) only (*N* = 17)

	Age 5–6						Age 9–10						Adults					
			
				HPD95 CIs						HPD95 CIs						HPD95 CIs		
															
Fixed Effects	*M* (β)	*SE*	*t*	Lower	Upper	*p*	*M* (β)	*SE*	*t*	Lower	Upper	*p*	*M* (β)	*SE*	*t*	Lower	Upper	*p*
(Intercept)	(0.49)	(0.19)	(2.60)	(0.10)	(0.82)	(.01)	−(0.09)	(0.16)	−(0.56)	−(0.37)	(0.20)	(.58)	−(0.39)	(0.12)	−(3.17)	−(0.68)	−(0.14)	(.00)
Rating for bare form	−0.17	0.11	−1.51	−0.35	0.13	.13	−0.04	0.13	−0.33	−0.33	0.23	.74	−0.15	0.19	−0.80	−0.51	0.26	.42
Reversibility	**0.57**	**0.23**	**2.44**	**0.10**	**1.13**	**.02**	0.01	0.16	0.08	−0.27	0.42	.93	−0.06	0.15	−0.42	−0.44	0.24	.67
Freq pre-empting forms	−0.01	0.06	−0.10	−0.12	0.13	.92	**−0.10**	**0.04**	**−2.46**	**−0.18**	**−0.02**	**.02**	0.00	0.03	−0.05	−0.09	0.08	.96
Freq bare form (ent)	−0.15	0.12	−1.25	−0.43	0.11	.22	0.03	0.09	0.30	−0.16	0.20	.77	−0.09	0.07	−1.25	−0.26	0.09	.21
Semantics—factor 1	0.18	0.23	0.77	−0.31	0.65	.44	**0.53**	**0.16**	**3.21**	**0.18**	**0.88**	**.00**	−0.09	0.15	−0.61	−0.44	0.25	.54

Random Effects	Var						Var						Var					
Verb	0.00						0.03						0.00					
Participant	*0.32*						0.28						0.00					
Residual	1.36						0.66						0.92					

*Note*: The fixed effects of Verb-Type and Frequency of the *un*- form are not included because all verbs are of the same type (zero), and hence, by definition, have an *un*- form frequency of 0.

**Table 4:** Mixed-effects models for un- verbs (verbs that DO take un-) only (*N* = 31)

	Age 5–6						Age 9–10						Adults					
			
				HPD95 CIs						HPD95 CIs						HPD95 CIs		
															
Fixed Effects	*M* (β)	*SE*	*t*	Lower	Upper	*p*	*M* (β)	*SE*	*t*	Lower	Upper	*p*	*M* (β)	*SE*	*t*	Lower	Upper	*p*
(Intercept)	(0.97)	(0.22)	(4.40)	(0.61)	(1.34)	(.00)	(1.15)	(0.10)	(11.57)	(0.96)	(1.36)	(.00)	(1.15)	(0.11)	(10.49)	(0.93)	(1.37)	(.00)
Freq *un*- form	0.02	0.04	0.50	−0.06	0.11	.62	**0.16**	**0.05**	**3.47**	**0.07**	**0.25**	**.00**	**0.28**	**0.06**	**4.78**	**0.16**	**0.40**	**.00**
Rating for bare form	0.09	0.07	1.32	−0.05	0.24	.19	**0.34**	**0.09**	**3.91**	**0.15**	**0.51**	**.00**	**0.35**	**0.08**	**4.28**	**0.20**	**0.51**	**.00**
Reversibility	0.05	0.13	0.38	−0.22	0.31	.70	−0.14	0.15	−0.91	−0.43	0.14	.36	−0.12	0.18	−0.65	−0.47	0.26	.52
Freq pre-empting forms	0.08	0.04	1.83	−0.01	0.18	.07	−0.07	0.05	−1.50	−0.15	0.02	.14	−0.01	0.06	−0.15	−0.12	0.11	.88
Freq bare form (ent)	−0.01	0.04	−0.34	−0.11	0.07	.73	**−0.12**	**0.05**	**−2.42**	**−0.21**	**−0.03**	**.02**	**−0.13**	**0.06**	**−2.30**	**−0.24**	**−0.03**	**.02**
Semantics—factor 1	0.17	0.10	1.65	−0.05	0.36	.10	**0.52**	**0.11**	**4.55**	**0.31**	**0.75**	**.00**	**0.39**	**0.14**	**2.76**	**0.13**	**0.67**	**.01**

Random Effects	Var						Var						Var					
Verb	0.02						0.09						0.21					
Participant	*0.70*						0.04						0.01					
Residual	0.95						0.84						1.29					

*Note*: The fixed effect of Verb-Type is not included because all verbs are of the same type (*un*-).

### 2.1. Age 5–6

The youngest children displayed no evidence of an effect for either pre-emption or entrenchment in any of the three analyses. Indeed, unlike the older groups, they displayed no frequency effects at all beyond simple existence/non-existence of the *un*- form (see Table 2, row labeled *Verb Type*). That is, they displayed an effect of verb type (*un*-/zero), but not *un*- or bare form frequency.

The lack of a pre-emption effect is to be expected under an account where children's own coinages (e.g., *unsqueeze) compete in memory with the equivalent adult forms (e.g., *release) for a protracted period (Clark & Clark, 1979; Clark, 1993; Maslen et al., 2004; Ramscar & Yarlett, 2007). The lack of an entrenchment effect is more surprising, given that a number of studies have observed such an effect with children of this age (Ambridge et al., 2008, 2009, 2011), and even younger (Brooks et al., 1999). One possibility is that these children are too young for a sufficient degree of entrenchment to have taken place. The other is that entrenchment is not an important factor in the retreat from error in this domain. Which of these two possibilities is more likely to be correct will become clear upon analysis of the findings for the two older groups.

Either way, these findings certainly constitute evidence against the claim that pre-emption and entrenchment effects will necessarily be observed before effects of verb semantics (e.g., Tomasello, 2003). Despite showing no effects of verb frequency, and with all the relevant control factors in place, the semantic feature ratings explained a significant proportion of unique variance, though only for the analysis including all verbs. Thus, 5–6-year olds use verb semantics, but it seems not yet pre-emption or entrenchment, to help them determine which verbs may and may not receive *un*- prefixation. However, at this age, semantics is not a significant predictor of the *degree* of either (a) unacceptability of ungrammatical *un*- forms or (b) acceptability of grammatical *un*- forms (though it may be possible to argue that this latter effect was moving in the predicted direction, although this was not yet significant, *p* = .10, n.s.).

At the same time, it is interesting to note that the younger children show a small but significant tendency to be influenced by reversibility (inappropriately so, when one considers adult performance). That is, younger children consider the reversibility of the action to predict the both (a) the likelihood that a verb may receive the prefix *un*- (Table 2) and (b) the degree of unacceptability that results when this prefix is used with verbs that may not in fact take *un*- (Table 3). Since this is not true for adults, this incorrect assumption may be the source of at least some of children's overgeneralization errors.

### 2.2. Age 9–10

The analysis including all verbs (Table 2) suggests that the 9–10-year olds displayed effects of both pre-emption and entrenchment. However, as argued above, these hypotheses make predictions about the relative unacceptability of ungrammatical *un*- forms only. Inspection of Table 3 reveals that the pre-emption measure is a significant predictor, but entrenchment is not. However, Table 4 reveals that an entrenchment effect is observed when restricting the analysis to *un*- forms that are “grammatical” (or at least attested in the BNC). This suggests that entrenchment is playing some role, but only for marginal cases where the *un*- form is attested, but is presumably less than fully grammatical for all speakers (e.g., the *un*- forms that appear just once in the corpus such as *unbandage, undelete,* and *unloosen*).

Thus, a fair conclusion is probably that 9–10-year olds—unlike the younger children—display some effect of both pre-emption and entrenchment. This pattern is predicted by an account under which pre-emption and entrenchment require a considerable amount of linguistic experience to begin to exert their effects.

The developmental pattern is not supported by an account under which entrenchment and pre-emption effects are observed before verb semantics: For the older children, the semantic predictor was significant under all three analyses. Thus, semantic properties predicted both the relative unacceptability of ungrammatical *un*- forms (Table 3) and the relative acceptability of grammatical *un*- forms (Table 4), as well as which forms may take *un*- in the first place (Table 2), even having controlled for whether they are attested in the BNC (note the significant effect of *Verb Type* in Table 2). Indeed, the effect of semantics is particularly impressive when one considers that, in the first analysis, all the control predictors except reversibility also explain a significant portion of variance. That is, 9–10-year olds give higher ratings for attested than unattested *un*- forms (verb type), for verbs that have higher frequency in *un*- form (*un*- form frequency) and for verbs that they deem more acceptable across the board (rating for bare form).

The finding of effects of pre-emption, entrenchment, and verb semantics raises the question of the relative importance of each factor. Are learners primarily using entrenchment and pre-emption to restrict their generalizations, noticing, but rarely using, correlations with semantics (e.g., Stefanowitsch, 2008)? Alternatively, are they using semantics as the primary determinant of *un*- prefixability (Pinker, 1989), perhaps turning to pre-emption or entrenchment for idiosyncratic items (e.g., Boyd & Goldberg, 2011)? Looking across all three analyses, and using the *t*-values as a rough estimate of effect size, it remains clear that the most important factor is whether a particular *un*- form has been encountered (or, at least, is attested in the BNC). This is unsurprising, as learners must clearly assume that forms that are used by native adult speakers are—by definition—grammatical. Moving beyond the control predictors, semantics would appear to be the most influential factor, with the summative effect of pre-emption and entrenchment not far behind. This suggests the need for an account of learning that yields all three effects, an issue to which we return in the discussion.

### 2.3. Adults

The most striking difference between the older children and adults is that the latter do not display a significant (or even marginal) effect of pre-emption in any analysis. Two related explanations seem feasible, one more methodological, one more theoretical. The methodological explanation is that adults were more binary in their judgments than children, with over 50% of responses either “completely unacceptable” or “completely acceptable” (see Fig. 2). Table 2 reveals that by far the largest predictor is verb type, suggesting that adults are mainly following a strategy of assigning 5 to *un*- forms they have heard and 1 to *un*- forms they have not heard. This leaves little variation to be explained by pre-emption (and also entrenchment and semantics).

The more theoretical explanation starts by asking *why* adults base their judgments mainly on attested usage, at the expense of pre-emption. A key point to bear in mind here is that the study used familiar, as opposed to novel, verbs. This means that, for adults, pre-emption may have reached asymptote. For example, children may still be learning that one says *release* rather than *unsqueeze or *empty* rather than *unfill, meaning that the availability of the pre-empting form matters. Having learned that *release* and *empty* are the relevant verbs to use in this situation, adults would not countenance *unsqueeze and *unfill, and the availability or otherwise of a pre-empting form to “block” the error is irrelevant.^10^

Consistent with this explanation is the finding that both the entrenchment and semantic predictors are significant for adults, but only in the overall analysis (Table 2) and the analysis restricted to attested *un*- forms (Table 4). Thus, there seem to be a number of verbs for which adults have definitively decided that the *un*- form is completely unacceptable. When looking only at these verbs (Table 3) neither entrenchment nor semantics influence the degree of unacceptability. However, these factors do play a role in determining the relative acceptability of attested *un*- forms, for example, by distinguishing between *un*- forms that are frequent and clearly display the appropriate semantics (e.g., *unlock, unleash, unpack,* and *undo*) and more marginal cases (e.g., *unbandage, uncrumple, undelete, unbelieve, unloosen,* and *untighten*).

If adults are basing their decisions mainly on attested usage, one might ask why an effect of verb semantics remains at all (indeed, this is the only one of the three effects that is observed at all ages^11^). An important consideration here is that semantics is a deeper explanation than pre-emption or entrenchment. Pre-emption and entrenchment work well as explanations of how children learn the way their language behaves (i.e., which verbs can occur in which generalizations). However, verb semantics is an explanation of why the language behaves in the way that it does (i.e., why some verbs are blocked from appearing in some generalizations in the first place). As an explanation of this deeper phenomenon, pre-emption and entrenchment are circular. Saying that *unfill is ungrammatical because we say *empty* instead is a perfectly good explanation of learning, but it is circular as an explanation of why this is the case. The deeper question is *why* we say *empty* instead of *unfill (or allowing both), and it is this question that a semantic account attempts to explain; the explanation here being that *fill* does not exhibit the requisite semantic properties. The finding that verb semantics significantly predicts the acceptability of the *un*- prefixed form at every age, even after controlling for other factors, would seem to support this explanation.

## 3. Discussion

This study used a grammaticality judgment paradigm to compare competing theoretical accounts of children's retreat from (or avoidance of) overgeneralization errors, focusing on the domain of reversative *un*- prefixation.

Perhaps, the most significant finding was that Whorf's (1956) semantic cryptotype hypothesis was supported for every age group. That is, adult ratings of the extent to which each verb exhibits semantic properties characteristic of the “covert class” of *un*- verbs was a significant predictor of the relative acceptability of that verb in *un*- form. This finding held even after controlling for (a) whether the verb is attested with *un*- in a suitable corpus (BNC), (b) corpus frequency of the *un*- form, (c) acceptability of the bare form, (d) reversibility, (e) frequency of the pre-empting forms, and (f) frequency of the bare form.

The second important finding was that the corpus frequency of the verb in bare form was, for the two older groups, a significant negative predictor of the acceptability of the *un*- form (in the main analysis and at least one by-verb-type analysis). This supports the entrenchment hypothesis, under which repeated presentation of a verb constitutes probabilistic evidence that its use in non-attested forms (here, with the prefix *un*-) is ungrammatical. For younger children, this effect did not reach significance, perhaps in part because they are inappropriately (given the findings for the older groups) influenced by reversibility.

Third, in support of the pre-emption hypothesis, the availability of alternative forms expressing the relevant meaning (e.g., *open* for *unclose)—operationalized as the total corpus frequency of the two most-suggested alternatives—was a significant negative predictor of the relative unacceptability of ungrammatical *un*- forms, but only for the older children. Again, the lack of an effect for the younger children may reflect a strategy of basing judgments on reversibility, or the fact that pre-emption requires a considerable degree of linguistic experience. For adults, it may well be the case that pre-emption has reached asymptote, leaving whether a verb is attested in *un*- form as the most important predictor.

In summary, while the developmental pattern is somewhat complex, it remains clear that any account of children's unlearning of *un*- prefixation errors must include some role for verb semantics, entrenchment, and pre-emption. What follows is one possible account of a learning mechanism designed to yield all three effects. The goal is an account under which, in the words of a reviewer (Danielle Matthews) “pre-emption, entrenchment, and semantic[s]…[are] seen as different aspects of one architecture and learning process.” It is important to stress that the account is “new” only to the extent that it combines elements of previous proposals and attempts to pin them down with more mechanistic precision than has previously been the case.

The starting point is an account originally outlined for overgeneralizations involving verb argument structure constructions such as the transitive causative (e.g., *The funny clown giggled Lisa; Ambridge et al., 2011), dative (e.g., *I said her no; Ambridge, Pine, & Rowland et al.^2012^), and locative (e.g., *I filled some juice into the cup; Ambridge, Pine, & Rowland, 2012; see Ambridge and Lieven [2011: 256–265] for a more detailed outline). The proposal is termed the *FIT* account because it emphasizes the importance of semantic *Fit* between *Item* and *Template* properties. The only additional assumption required to extend this account to the domain of *un*- prefixation is that *un*- prefixation involves a morphological construction—*un-[VERB]*—that is analogous to argument-structure constructions such as the transitive causative [SUBJECT] [VERB] [OBJECT]. Although, from some perspectives, this assumption may seem controversial, the uniform representation of syntactic and morphological constructions is assumed by all construction grammar approaches (and, indeed, is listed by Croft & Cruse, 2004, as one of three “essential principles” of this approach).

The basic assumption of the account is that learners acquire constructions (at whatever level) by abstracting across strings in the input (e.g., across forms such as *untie*, *unwrap,* and *unbutton* to form a *un*-[VERB] construction). That is, the proposal is an exemplar-based model that “recognizes input and generates outputs by analogical evaluation across a lexicon of distinct memory traces of remembered tokens of speech” (Gahl & Yu, 2006: 213; see also Bod, 2009). Each slot in each construction (here [VERB]) probabilistically exhibits the semantic properties shared by the items that appeared in this position in the utterances that give rise to the construction (Dabrowska & Lieven, 2005; Goldberg, 1995; Langacker, 2000; Naigles, Fowler & Helm, 1992; Naigles, Gleitman & Gleitman, 1993; Suttle & Goldberg, 2011).^12^ For example, the [VERB] slot in the *un*-prefixation construction will exhibit properties such as *covering, enclosing,* and *change of state* (Whorf, 1956).

The acceptability of a particular form (e.g., *unsqueeze) reflects the semantic compatibility of the verb and the construction slot into which it is inserted (e.g., Bowerman, 1981), via a process known variously as “fusion” (Goldberg, 1995: 50), “unification” (Kay & Fillmore, 1999), or “elaboration” (Langacker, 2000). This assumption is designed to explain the phenomenon that acceptability is a graded, not an absolute, phenomenon (as observed in this study).

So far, this proposal amounts to little more than a re-framing of Whorf's (1956) semantic cryptotype account in the terminology of construction grammar. However, this re-framing buys us two things. First, it allows the retreat from *un*- prefixation error to be treated in exactly the same way as the retreat from argument-structure overgeneralization errors (e.g., errors involving the transitive causative). Second, it allows for the explanation of pre-emption and entrenchment effects as statistical learning procedures that operate over this semantic fit process (as opposed to simply as separate additional mechanisms).

For the following example, let us assume that a speaker wishes to convey a particular message: the reversal of an action of appearance. We further assume that the speaker has a lexicon consisting of stored verbs and constructions, each with a resting activation level proportional to the frequency with which it has been previously encountered. The production mechanism must select a verb and a construction to express the desired message. Verb and construction pairs compete for activation (MacWhinney, 2004), which is determined by the following three factors:

1. *Relevance:* Verbs and constructions receive an activation boost proportional to the extent to which each expresses the meaning to be conveyed. For this example, verbs that will receive a large boost include *appear, materalize,* and *vanish*. Constructions that will receive a large boost include *un*-[VERB], *dis*-[VERB], and *vanish* (which is conceptualized as a fully lexically specified construction). However, relevance is conceptualized as gradient rather than all-or-nothing in nature; verbs and constructions that are less—though still somewhat—relevant (e.g., *leave, go*, *de-[VERB]*) will receive a smaller boost.
2. *Item-in-construction frequency:* The activation boost received by highly relevant verbs (e.g., *appear, materialize, vanish*) spreads to the constructions in which those verbs have been previously encountered, in proportion to the frequency with which the verb has appeared in each. For example, *appear* will boost the activation of *dis-[VERB]* but not *un-[VERB]* or *de-[VERB]*, while *materialize* will boost only f*de-[VERB]*. For fully lexically specified constructions (e.g., the lexical item *vanish*), item-in-construction frequency is equivalent to simple lexical frequency. Importantly, even constructions that are not relevant to the speaker's message will receive this activation boost (e.g., *appear* will also boost the activation of *re-[VERB]* and the simple intransitive construction *[SUBJECT] [VERB]*, among many others). However, these constructions are extremely unlikely to selected for use, as activation depends on some function of item-in-construction frequency and relevance (on which they score very low), as well as *fit*.
3. *Fit:* Verb and construction pairs receive an activation boost proportional to the fit between semantic properties of the lexical item (e.g., *appear*) and the relevant construction slot (e.g., *un*-VERB; *dis*-VERB), as outlined above (for other constructions, *fit* may be defined in terms in phonological or pragmatic properties; e.g., Ambridge & Lieven, 2011). For fully lexically specified constructions (e.g., *vanish*), fit is trivially perfect. For constructions with abstract slots (e.g., *un-[VERB]*), fit is a graded phenomenon (as demonstrated in this study).^13^

As the outcome of this process, the verb is inserted into the relevant slot (or a fully lexically specified construction is output) to yield the utterance. When there is sufficient overlap between the properties of the slot and its filler, a grammatical form results (e.g., *un-[wrap]*; *dis-[appear]*). An ungrammatical form (e.g., *unappear) results when the child inserts a lexical item into a slot with which—from the adult viewpoint—it is less than optimally compatible in terms of its semantic properties. There are (at least) three scenarios in which this can occur (these are not intended to be mutually exclusive):

1. The child has yet to fully acquire the relevant properties of either the particular slot or the particular lexical item concerned (or both). For this example, the child may have yet to learn that the VERB slot in the *un*-VERB construction exhibits the properties of *change of location*, *A and B are separable*, *circular movement,* etc., and/or that the item *appear* does not exhibit these properties.
2. The child may be aware of the suboptimal fit between the slot and its filler, but may have yet to acquire a construction with a more appropriate slot. For example, she might insert *appear* into the *un*-VERB construction, yielding *unappear, because she has yet to acquire the competing *dis*-VERB (or the fully lexically specified construction *vanish*).
3. The child may be aware of the suboptimal fit between the slot and its filler, but this particular verb + construction pair nevertheless wins the competition for activation due to an usually high degree of relevance (and/or high resting activation level of either the verb or the construction). For example, children who produce overgeneralization errors such as *unhate or *unsqueeze may be aware, to some extent, of the semantic mismatch between verb and construction semantics, but choose this construction anyway because it so perfectly fits the desired message (relevance) and/or because *hate, squeeze,* and *un-[VERB]* are more frequent than alternatives like *forgive* or *release*. Relevance also seems to trump fit in adult coinages that are intended to be humorous (e.g., *Ungrow up*; the slogan for a recent advertising campaign for a brand of chocolate).

The mechanism for the retreat from error is learning more about the semantics of the individual lexical item (here the verb) and the relevant slot (here, the *[VERB]* slot in the *un-[VERB]* construction), to the point where generalizations that would involve a high degree of incompatibility between one and the other are outcompeted by more felicitous pairings. It is beyond the scope of the present proposal to outline precisely how these semantic properties are learned. At the level of individual verbs, the assumption is simply that children notice the commonalities between all of the subtly different events to which a particular verb (e.g., *squeeze*) is used to refer, with properties that vary across events (e.g., the particular object being squeezed) eventually ignored (presumably something like this process is assumed by all accounts of verb learning, and indeed of word learning in general). Similarly, with regard to learning the semantic properties of construction slots, the account assumes no particular semantic primitives. The properties of the slot are simply a weighted average of the properties of those items that have appeared in this position in input utterances, be those properties semantic, phonological, or something else (see Ambridge & Lieven, 2011: 261–262; Suttle & Goldberg, 2011).

This mechanism yields pre-emption effects as a function of relevance and item-in-construction frequency. For the present example (reversal of an appearance action), the constructions *un-[VERB]*, *dis-[VERB],* and the fully lexically specified construction *vanish* all score high on relevance. Thus, the higher the frequency of alternatives such as *dis-[appear]*, *vanish*, the greater the extent to which these will be preferentially activated over *unappear. Thus, this mechanism yields the frequency-sensitive pre-emption effect observed in this study (for the older children) and many others.

The mechanism also yields entrenchment effects as a result of item-in-construction frequency. Recall that relevant verbs (e.g., *appear*) boost the activation of all constructions in which they appear—even those that score low on relevance (e.g., [SUBJECT] [VERB])—in proportion to their frequency of co-occurrence. Thus, a verb that occurs frequently in constructions other than *un-[VERB]* (i.e., that has high “bare form” frequency) will preferentially activate these constructions at the expense of the *un-[VERB]* construction, thus making overgeneralization errors less likely. Thus, this mechanism yields the frequency-sensitive entrenchment effect observed in this study (for the two older groups) and many others.

Finally, this mechanism yields semantic effects, both (a) at a “verb class” level, because verbs with similar meanings will cluster into (descriptive) classes that are in/compatible with a similar range of constructions, and (b) at a continuous level, as observed in this study; a pattern that is potentially problematic for discrete-class-based accounts.

It is important to emphasize that this account is not intended to replace or provide an alternative to entrenchment, pre-emption, or the formation of semantic cryptotypes. Indeed, in one sense, this proposal constitutes simply a redescription of these effects. However, the goal of this proposal is to redescribe each phenomenon in such a way that allows for their implementation in a single learning mechanism, with sufficient precision to allow for the implementation of this account as a computational model.

Computer-modeling work may help to clarify an important outstanding issue. Currently, the verbal account outlined above makes no specific claims with regard to the relative contributions of fit, relevance, and item-in-construction frequency at each age. Consequently, the account does not currently explain the observed changes in the relative magnitude of pre-emption, entrenchment, and verb semantic effects with development. Implementing this account as a computational model will necessitate the specification of the relative contributions of fit, relevance, and item-in-construction frequency, and the resulting model will yield quantitative predictions that can be tested against experimental data.

Future experimental work should test more directly the claim of the present account that children's errors are a consequence of non-adultlike knowledge of verb or slot semantics, and the developmental prediction that such errors will cease at the point at which this knowledge is acquired.

In the meantime, there exists one study that provides preliminary support for at least part of this proposal, with regard to the locative constructions. The VERB slot in the figure- (or contents-) locative construction ([AGENT] [VERB] [CONTENTS] into [CONTAINER]) is associated with the semantic property of manner of motion (e.g., *pour, dribble*; as in *Lisa poured water into the cup*). Conversely, the VERB slot in the ground- (or container-) locative construction ([AGENT] [VERB] [CONTAINER] with [CONTENTS]) is associated with the semantic property of state-change (e.g., *fill, soak*; as in *Lisa filled the cup with water*). Children occasionally make overgeneralization errors where ground-only verbs (e.g., *fill*) are overgeneralized into the figure-locative construction (e.g., *Lisa filled water into the cup). Under the present account, one source of such errors will be immature verb knowledge: If children think *fill* denotes “manner of motion” rather than “state change,” they will experience no lack of fit between the semantic properties of this lexical item and the VERB slot in the figure-locative construction. Accordingly, Gropen, Pinker, Hollander, and Goldberg (1991) found that children who produced overgeneralization errors with *fill* (e.g., *Lisa filled water into the cup) judged pouring events where a glass ended up only three-quarters full to be perfectly good examples of “filling.” This suggests that, for these children, *fill* had the semantic properties of manner of motion (i.e., was similar in meaning to adult *pour*), as opposed to state-change, and hence was perfectly compatible with the semantic properties of the VERB slot in the figure-locative construction.

Future studies should build on this paradigm in three ways. First, such investigations should be extended to the *un*- prefixation, transitive causative and dative constructions (and indeed, any constructions for which overgeneralization errors are observed, in any language). Second, assuming that the obvious methodological challenges can be overcome, children should be asked to rate individual verbs for their semantic properties—as adults did for this study—to investigate the relationship between knowledge of verb semantics and ratings of overgeneralization errors in more detail. Finally, future studies should test the prediction that errors arise as consequence of immature knowledge of slot—as opposed to filler—semantics. An experimental approach that may be useful here is the novel-construction-learning paradigm of Casenhiser and Goldberg (2005). Under this paradigm, children are taught novel argument-structure constructions characterized by non-English word order (and sometimes case-marking morphemes) associated with a particular meaning (e.g., appearance). The present account predicts that children should initially produce and accept uses of verbs that are inconsistent with this meaning but cease to do so as they gradually learn that the VERB slot exhibits the semantic property of appearance.

Thus in conclusion, although the account outlined here may need refining further, it is clear that—alone—entrenchment, pre-emption, or semantic-verb-class formation are insufficient to account for the retreat from overgeneralization error. Rather, any successful proposal will almost certainly have to include a role for all three factors, with verb semantics operating in a more continuous and probabilistic manner than proposed under the semantic verb class hypothesis.

## Notes

1 It is sometimes claimed (e.g., Pinker, 1989) that children make analogous overgeneralization errors with passives. However, as an anonymous reviewer pointed out, none of the attested errors (e.g., **I'm making her picture scribbled*; Bowerman, 1988) are overextensions of an attested generalization in the same way as for the other constructions discussed here. That is, children do not seem to make overgeneralization errors such as *The book cost $5→*$5 was cost by the book* on analogy with pairs such as *The bus hit John→John was hit by the bus*. For the passive, then, the question is probably not “how do children retreat from attested errors?” (as for the other constructions discussed here), but “why is this particular construction apparently immune to error?”. This, of course, is a question for another paper.

2 Indeed, it would be interesting to run training studies in this and related domains to investigate whether, as predicted, the amount of negative evidence that children receive is a negative predictor of error rates (and/or rated grammaticality).

3 A reviewer (Danielle Matthews) suggested that it is not quite accurate to set up pre-emption, entrenchment, and semantics as separate mechanisms; “Rather, they might be seen as different aspects of one architecture and learning process.” While I agree that what is needed is an account under which the three processes are an outcome of a single learning mechanism, I would argue that previous proposals have generally treated them as separate from one another. For example, Stefanowitsch (2008), Goldberg (2008), Stefanowitsch (2011), and Boyd and Goldberg (2011) explicitly pit pre-emption against entrenchment, and conduct studies designed to differentiate between the two, while Pinker's (1989) semantic verb class hypothesis makes no mention of either. Even a proposal designed to incorporate all three (Tomasello, 2003: 180) differentiates the processes developmentally, claiming that “entrenchment works early…and semantic subclasses begin to work later, perhaps not until about 4;6 or so.” Similarly, Perfors, Tenenbaum, and Regier (2011: 636) argue that “although semantic information is ultimately used, syntactic information [i.e., entrenchment/pre-emption] may be more important initially.”

4 A particularly sophisticated variant of this hypothesis (Stefanowitsch, 2008) posits that learners also take into account the overall frequency of the target construction. Thus, a potential generalization is deemed ungrammatical if the occurrence of the verb (e.g., *fall*) in the target construction (here, the transitive) is significantly less frequent in the input than would be expected, given the independent frequencies of the verb and the construction. However, when focusing on a single construction—as in this study—construction frequency is constant, meaning that Stefanowitsch's “negative entrenchment” hypothesis is exactly equivalent to the standard entrenchment hypothesis.

5 There would certainly seem to be some cases where verb behavior is purely idiosyncratic, and has no semantic basis (e.g., *manage to do; succeed in doing* vs. **succeed to do; *manage in doing*). The question is whether these are simply low-level lexical restrictions that are qualitatively different from the more abstract verb argument structure constructions discussed by Pinker (1989)—for example, [SUBJECT] [VERB] [OBJECT]—and, to some extent, the domain investigated in this study.

6 Although Whorf's particular proposal is specific to English, somewhat similar semantic constraints apply to the historically related Dutch prefix *ont*- (Lieber & Baayen, 1993), and to the Spanish prefix *des*- (Aguado-Orea, p.c., 11/08/2011). More important, the more general claim that certain generalizations apply only to items with particular semantic properties is likely true at some level for most languages. Furthermore, to the extent that few linguistic regularities are entirely predictable, it is probably fair to say that many will share this “cryptotype” nature.

7 It is important to clarify that since there was no longitudinal element to the study, it is not possible to observe how pre-emption, entrenchment, and semantics lead to the retreat from overgeneralization at the individual level. Nevertheless, the study bears directly on the issue of the retreat from overgeneralization: By selecting verbs that vary with regard to their values on the pre-emption, entrenchment and semantic measures, we can investigate longitudinal change by comparing snapshots at points in development. For example, the assumption is that comparing a verb with a frequency of 10 to a verb with a frequency of 50—after controlling for semantics and other factors—is essentially the same as comparing the same verb after 10 and 50 occurrences. Indeed, an exactly analogous assumption at the level of participants as opposed to items is tacit in all studies that compare multiple age groups at a single point in time, as opposed to testing the same participants repeatedly across development.

8 An anonymous reviewer objected that the training examples do not illustrate graded acceptability as “the only cases where judgments can be clearly in terms of preference are forms like *spilt* versus *spilled* where both forms are allowed, yet one tends to be preferred in a given variety of English.” In fact, though, the question of whether graded acceptability can arise in the absence of two competing forms is an empirical one; and the findings of this study strongly suggest that it can. In any case, whatever their merits and shortcoming, the training examples seemed to successfully serve their purpose of training participants to use the whole of the scale (see the descriptive statistics at the beginning of the results section).

9 A reviewer asked for clarification of why *un*- forms were prohibited. The reason is that the task was designed to yield possible forms that are *alternatives to*, and hence may pre-empt, particular *un*- forms.

10 An anonymous reviewer objected to this conclusion saying “attested use cannot really explain the difference between 9 and 10-year olds and adults,” “since adults (like children) readily coin new verbs with the *un*- prefix (with appropriate semantics).” However, given the restricted stimulus set of this study, the zero verbs did not have appropriate semantics, and hence were not available for coinage in *un*- form. Thus, there is no contradiction in arguing that adults were performing this particular rating task largely on the basis of attested usage, and acknowledging their capacity for productive generalization elsewhere. The claim is not that pre-emption has reached asymptote in adults in general, just for these particular verbs. It would seem likely that if adults were presented with novel verbs, they would make use of both semantics and pre-empting alternatives when determining the grammaticality or otherwise of the *un*- form (see Boyd & Goldberg, 2011, for evidence of the latter).

11 It is important to emphasize that although semantics is not always a significant predictor for both subsets (*un*- and zero verbs), it is always significant in the analysis that includes all verb types (even when including verb type as a predictor). This is the most appropriate analysis as, unlike pre-emption and entrenchment, the semantic account makes predictions that collapse across verb type.

12 One study that predicted, but did not find, an effect of construction-slot semantics is that of Matthews and Bannard (2010). These authors found that 2- and 3-year-old children showed no increased ability to repeat a familiar string when the relevant slot-filler was semantically similar to the corresponding filler in a high frequency (and hence presumably well-learned) string. For example, considering the familiar string *a piece of toast,* children made just as many errors when repeating *a piece of meat* as *a piece of brick*. However, an important difference from this study is that the semantic properties of the fillers (e.g., *brick*), though *atypical* of the semantic properties of the slot were not *incompatible* with them.

13 It is important to emphasize that semantic fit is not a broad-brush prohibition against utterances that “make no sense.” The paradox to be explained lies in the fact that overgeneralization errors such as **I unsqueezed it* or **The clown laughed the man* do make sense; that is why children produce them. Yet somehow, speakers must learn that, nevertheless, such utterances are regarded as ungrammatical by adult speakers. Neither is the present account an alternative to a straw-man position that “semantics is unimportant” when determining the constructions in which verbs may and may not appear. Rather, it is a particular account of *how* semantics exerts its effects; an account that is different in many important respects to rival semantics-based accounts such as that of Pinker (1989).

## Appendix: Training sentences (with expected responses) and test sentences

Training sentences

1. Lisa broke the cup (5/5).
2. Lisa breakded the cup (1/5).
3. Bart spilled soup onto his shirt (suggested score 4/5 as *spilt* is preferred in British English).
4. Homer eated the ice cream (suggested score 2/4 as more acceptable than *breakded*).
5. Homer drap water into the cup (suggested score 1/5—over-irregularization of *drip*).
6. Bart sticked the stickers onto his shirt (suggested score 2–3/5; better than *breakded/drap*).
7. Lisa spreaded butter onto the bread (suggested score 4/5 as spread is the correct form, though many find spreaded acceptable).

Test sentences (note that participants were asked and trained to rate the acceptability of the individual verb forms, with the sentence merely providing context).

**Table:**

Bart (un)buttoned his shirt	Lisa (un)bandaged her arm
Bart (un)chained the dog to/from a post	Lisa (un)believed in unicorns
Bart (un)embarrassed everyone*	Lisa (un)froze the ice lolly
Bart (un)filled the balloon	Lisa (un)leashed the dog
Bart (un)hooked the picture on/from the wall	Lisa (un)locked the door
Bart (un)laced his shoes	Lisa (un)opened the box
Bart (un)masked the cat	Lisa (un)rolled (up) the newspaper
Bart (un)pulled the cord	Lisa (un)squeezed the sponge
Bart (un)went to the hospital	Lisa (un)tied her shoelaces
Homer (un)asked a question	Marge (un)allowed Bart some chocolate
Homer (un)bent the metal bar	Marge (un)closed the door
Homer (un)buckled his belt	Marge (un)crumpled the paper
Homer (un)came home	Marge (un)deleted the email
Homer (un)corked the bottle	Marge (un)gave Bart a cookie
Homer (un)did his tie	Marge (un)pressed the lever
Homer (un)fastened his seatbelt	Marge (un)put the book on/off the table
Homer (un)latched the gate	Marge (un)reeled the cotton
Homer (un)lifted his arms	Marge (un)released the bees
Homer (un)loosened his tie	Marge (un)removed the television
Homer (un)packed his case	Marge (un)screwed the top onto/from the container
Homer (un)sat on the dog	Marge (un)straightened the picture
Homer (un)snapped the Lego bricks together/apart	Marge (un)zipped her coat
Homer (un)stood on the box	
Homer (un)tightened the screws	
Homer (un)veiled the bride	
Homer (un)wrapped the present	

* *Note*: Some verbs that are ungrammatical in verbal *un*- prefixed form (e.g., **Bart unembarrassed everyone*) may appear in an adjectival past-participle *un*- form with the general meaning of “not,” with no reversal implied (e.g., a person may be described as *unembarrassed*, meaning simply “not embarrassed”). According to Clark et al. (1995: 635) and Marchand (1969: 205), the two *un*- prefixes are etymologically distinct. The non-reversative prefix (as in *unembarrassed*) had the form *ond*- in Old English but merged with the existing reversative *un*- prefix, likely due to the phonological and conceptual similarity between the two. Of course, it would seem likely that the existence of an adjectival *un*- form will increase the rated acceptability of a verbal *un*- form to which it is—on the surface—identical. This is controlled for in the regression, in which the surface frequency of each *un*- form in the British National Corpus is entered as a control predictor.
